# The fixed-dose combination of pertuzumab and trastuzumab for subcutaneous injection in Chinese patients with HER2-positive early breast cancer: primary analysis of the phase III, randomized FDChina study

**DOI:** 10.1007/s10147-025-02935-7

**Published:** 2026-05-07

**Authors:** Tao Huang, Zhimin Fan, Yongsheng Wang, Xi Yan, Hongjian Yang, Shu Wang, Da Pang, Huiping Li, Haibo Wang, Cuizhi Geng, Liang Huang, Yaqing Sun, Bei Wang, Guofang Sun, Asna Siddiqui, Eleonora Restuccia, Zhimin Shao

**Affiliations:** 1 Breast and Thyroid Surgery, Union Hospital, Tongji Medical College, Huazhong University of Science and Technology, Wuhan, 430022 China; 2 Department of Breast Surgery, Jilin University, Changchun, 130021 China; 3 Breast Cancer Center, Shandong Cancer Hospital, Jinan, 250117 China; 4 Medical Oncology, West China Hospital, Sichuan University, Chengdu, 610041 China; 5 Breast Surgery, Zhejiang Cancer Hospital, Hangzhou, 310022 China; 6 Breast Center, Peking University People’s Hospital, Beijing, 100044 China; 7 Department of Breast Surgery, Harbin Medical University Cancer Hospital, Harbin, 150040 China; 8 The Key Laboratory of Carcinogenesis and Translational Research (Ministry of Education), Department of Breast Oncology, Peking University Cancer Hospital and Institute, Beijing, 100142 China; 9 Department of Breast Cancer, Affiliated Hospital of Qingdao University, Qingdao, 266000 China; 10 Breast Center, The Fourth Hospital of Hebei Medical University, Shijiazhuang, 050035 China; 11 Department of Breast Surgery, Fudan University Shanghai Cancer Center, Shanghai, 200032 China; 12 Product Development China, Roche (China) Holding Co. Ltd, Shanghai, 201203 China; 13 Clinical Pharmacology, Genentech Inc., San Francisco, CA 94080 USA; 14 Product Development Clinical Oncology, Roche Products Limited, Welwyn Garden City, AL7 1TW UK; 15 Product Development Oncology, F. Hoffmann-La Roche Ltd, Basel, 4070 Switzerland; 16 Breast Cancer, Fudan University Shanghai Cancer Center, Shanghai, 200032 China

**Keywords:** Chinese patients, Fixed-dose combination, HER2-positive early breast cancer, Pertuzumab, Subcutaneous, Trastuzumab

## Abstract

**Background:**

Neoadjuvant fixed-dose combination of pertuzumab and trastuzumab for subcutaneous injection (PH FDC SC) demonstrated non-inferior cycle 7 pertuzumab and trastuzumab serum trough concentrations (C_trough_), and similar total pathological complete response (tpCR) rates and safety, to intravenous pertuzumab plus trastuzumab (P + H IV) in HER2-positive early breast cancer (eBC). In the FDChina study (NCT04024462), we assessed neoadjuvant–adjuvant PH FDC SC vs. P + H IV in Chinese patients with HER2-positive eBC.

**Methods:**

Patients received four doxorubicin (60 mg/m^2^) plus cyclophosphamide (600 mg/m^2^), then four docetaxel (75–100 mg/m^2^) cycles, every 3 weeks. Patients were randomized 1:1 to PH FDC SC (loading: 1200 mg pertuzumab/600 mg trastuzumab; maintenance: 600 mg/600 mg) or P + H IV (loading: 840 mg/8 mg/kg; maintenance: 420 mg/6 mg/kg) alongside docetaxel before surgery. Patients then continued HER2-targeted therapy for 14 cycles. Co-primary non-inferiority endpoints: cycle 7 pertuzumab and trastuzumab C_trough_. Secondary endpoints: tpCR, long-term efficacy, safety.

**Results:**

The lower bounds of the 90% confidence intervals of pertuzumab and trastuzumab cycle 7 geometric mean ratios (0.99 and 1.44, respectively) exceeded the pre-specified non-inferiority margin (0.8). tpCR rates were 55.6% for PH FDC SC vs. 56.4% for P + H IV. Grade ≥ 3 adverse events occurred in 72% vs. 69% of patients.

**Conclusion:**

The study met the co-primary endpoints of non-inferiority of cycle 7 serum C_trough_ for pertuzumab and trastuzumab for PH FDC SC vs. P + H IV. tpCR rates and safety were comparable between arms. PH FDC SC may be a viable treatment option for Chinese patients with HER2-positive eBC.

**Supplementary Information:**

The online version contains supplementary material available at 10.1007/s10147-025-02935-7.

## Introduction

The addition of intravenous (IV) pertuzumab to trastuzumab and chemotherapy significantly improved progression-free and overall survival in patients with HER2-positive metastatic breast cancer (mBC) in the CLEOPATRA trial, significantly improved pathologic complete response (pCR) rates in the neoadjuvant setting in the NeoSphere trial, and significantly and clinically meaningfully improved invasive disease-free survival in the adjuvant curative setting in patients at high risk of recurrence in the APHINITY trial [1–4]. IV pertuzumab plus trastuzumab (P + H IV) and chemotherapy is a standard treatment component for patients with HER2-positive early BC (eBC) or mBC [5, 6]. Compared with IV infusion, subcutaneous (SC) injection has several advantages, including reduced medical resource use and administration time; it is also less invasive, preferred by patients, and more satisfactory for healthcare professionals [7–12]. A fixed-dose combination of pertuzumab and trastuzumab for SC injection (PH FDC SC; pertuzumab, trastuzumab, and hyaluronidase-zzxf) has been approved by the US Food and Drug Administration and the European Medicines Agency [13, 14]. In the phase III FeDeriCa study, PH FDC SC demonstrated non-inferior cycle 7 pertuzumab and trastuzumab serum trough concentrations (C_trough_) to P + H IV in the neoadjuvant–adjuvant setting, with comparable total pCR (tpCR) rates and similar safety profiles between PH FDC SC and P + H IV [15]. However, the proportion of Asian patients in FeDeriCa was low (21% per arm) [15] and it is essential to prospectively assess treatments shown to benefit patients in global clinical trials in specific racial groups. Therefore, the phase III FDChina study (NCT04024462) was designed to investigate PH FDC SC vs. P + H IV in Chinese patients with HER2-positive eBC in the neoadjuvant–adjuvant setting. We report the primary analysis.

## Patients and methods

### Study design and patients

**Fig. 1:**
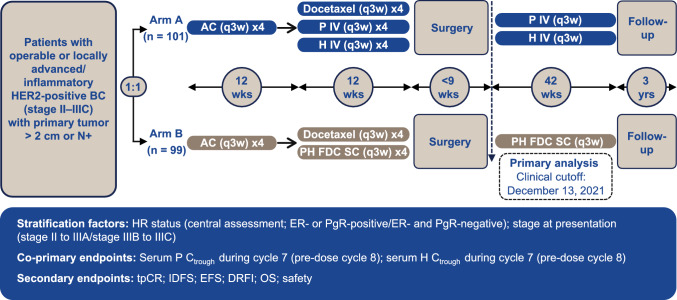
Study design. *AC* doxorubicin plus cyclophosphamide, *BC* breast cancer, *C*_*trough*_ trough concentration, *DRFI* distant recurrence-free interval, *EFS* event-free survival, *ER* estrogen receptor, *H* trastuzumab, *HR* hormone receptor, *IDFS* invasive disease-free survival, *IV* intravenous, *N* + node-positive, *OS* overall survival, *P* pertuzumab, *PgR* progesterone receptor, *PH FDC SC* fixed-dose combination of pertuzumab and trastuzumab for subcutaneous injection, *q3w* every 3 weeks, *tpCR* total pathological complete response, *wks* weeks, *yrs* years

FDChina was a randomized, multicenter, open-label, non-inferiority study. The design is shown in Fig. 1. Eligible patients were ≥ 18 years of age and had locally advanced, inflammatory, or early-stage BC (Stage II–IIIC) with centrally confirmed HER2-positive (immunohistochemistry 3 + and/or *HER2* amplification by in situ hybridization with a ratio of ≥ 2 for the number of *HER2* gene copies to the number of signals for chromosome 17) and hormone receptor (HR) status, with a primary tumor > 2 cm or node-positive disease, an Eastern Cooperative Oncology Group performance status (ECOG PS) of 0/1, and left ventricular ejection fraction (LVEF) of ≥ 55%. Patients must have agreed to undergo mastectomy or breast conserving surgery after neoadjuvant therapy. Patients previously treated for BC with any systemic therapy or radiation therapy were excluded.

Patients received four doxorubicin (60 mg/m^2^) plus cyclophosphamide (600 mg/m^2^) cycles, then four docetaxel (75–100 mg/m^2^) cycles, every 3 weeks. Patients were randomized 1:1 via interactive voice- or web-based response systems using a permuted-blocks randomization procedure to PH FDC SC (loading: 1200 mg pertuzumab plus 600 mg trastuzumab; maintenance: 600 mg pertuzumab plus 600 mg trastuzumab) or IV pertuzumab (loading: 840 mg; maintenance: 420 mg) plus trastuzumab (loading: 8 mg/kg; maintenance: 6 mg/kg) every 3 weeks, alongside docetaxel. Patients then had surgery before continuing HER2-targeted therapy for 14 cycles. Treatment was discontinued in cases of investigator-assessed disease progression or recurrence, or unmanageable toxicity. Stratification factors were HR status (estrogen receptor [ER]- or progesterone receptor [PgR]-positive vs. ER- and PgR-negative) and stage at presentation (Stage II–IIIA vs. Stage IIIB–IIIC).

The study was performed in conformance with Good Clinical Practice guidelines and the Declaration of Helsinki. The protocol was approved by the institutional review board or ethics committee at each study site. All patients provided written, informed consent.

### Endpoints

The co-primary endpoints were non-inferiority of the cycle 7 pertuzumab and trastuzumab serum C_trough_ (pre-dose cycle 8) of PH FDC SC vs. P + H IV.

Pre-specified secondary endpoints included tpCR (eradication of invasive disease in the breast and axilla [ypT0/is ypN0], according to local pathologist assessment) and safety. Incidence and severity of adverse events (AEs) were assessed until treatment completion or discontinuation per National Cancer Institute Common Terminology Criteria for Adverse Events version 4 [16]. Primary cardiac safety endpoints were incidence of symptomatic ejection fraction decreases (heart failure) of New York Heart Association (NYHA) class III or IV and a drop in LVEF of ≥ 10% from baseline to an absolute value of < 50%, and cardiac death. The incidence of asymptomatic or mildly symptomatic left ventricular systolic dysfunction (NYHA class II heart failure) was defined as a confirmed decrease in LVEF of ≥ 10% to an absolute LVEF of < 50%.

Secondary long-term efficacy endpoints (invasive disease-free survival, event-free survival, distant relapse-free survival, and overall survival) will be reported separately when the data are mature.

### Procedures

Blood samples for pharmacokinetic (PK) assessments were collected on dosing and specified non-dosing days during cycles 5–8. PK samples on dosing days were collected before administration of HER2-targeted therapy. Additional samples were collected within 15 min of HER2-targeted therapy and before chemotherapy for patients receiving IV infusion. Tumor response assessments were performed at screening and before each new therapy cycle.

### Statistical methods

Sample size calculations were based on the coefficient of variation for the C_trough_ of trastuzumab observed from previous eBC and mBC studies. With an assumed coefficient of variation of 60%, ≥ 80 patients per arm were needed to demonstrate C_trough_ non-inferiority (using a one-sided 95% confidence interval [CI]) with a power of 80% if the true means of the two formulations did not differ (the geometric mean ratio [GMR] = 1). The planned sample size was increased to 200 (100 patients per arm), as it was expected that 80% of patients would be PK-evaluable.

The primary analysis was performed in the per-protocol PK population; patients were excluded if they deviated significantly from the protocol or if relevant data were unavailable or incomplete. Safety was assessed in all patients who received ≥ 1 dose of study medication.

A non-inferiority margin of 0.8 was applied for the co-primary PK endpoints; non-inferiority was concluded if the lower bound of the two-sided 90% CIs of the GMR (C_trough SC_/C_trough IV_) was ≥ 0.8. A hierarchical approach was applied to control the overall type I error rate at a one-sided 5% significance level; cycle 7 trastuzumab C_trough_ was tested only if non-inferiority was concluded for cycle 7 pertuzumab C_trough_.

The study was not powered to assess non-inferiority of tpCR and no tpCR hypotheses were pre-specified.

## Results

### Patients

A total of 200 patients were randomized between February 5, 2020, and June 15, 2021. The clinical cutoff date was December 13, 2021. There were 99 patients in the PH FDC SC arm and 101 patients in the P + H IV arm in the intention-to-treat (ITT) population, and 100 patients per arm in the safety population. One patient who was assigned to P + H IV was mistakenly given PH FDC SC at cycle 5, so was included in the PH FDC SC arm in the safety population. Per-protocol PK populations for pertuzumab and trastuzumab were 89 patients per arm, and excluded the patient who was assigned to P + H IV but mistakenly took PH FDC SC (a pre-defined PK protocol violation).

**Fig. 2:**
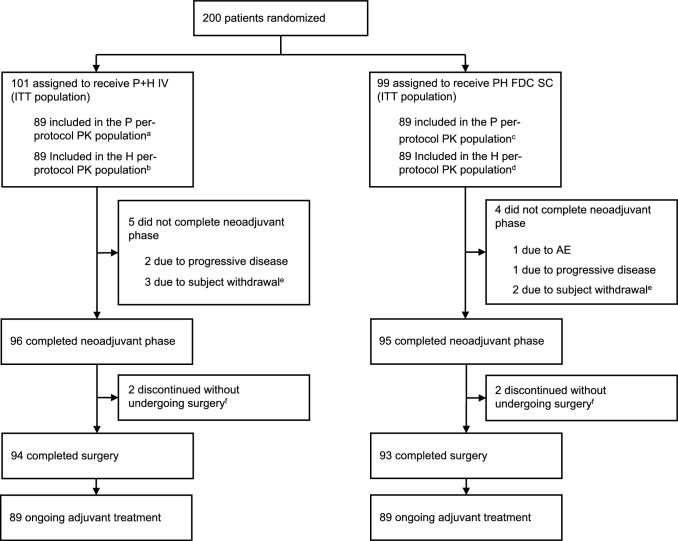
Patient disposition. *AE* adverse event, *C*_*trough*_ trough concentration, *H* trastuzumab, *ITT* intention to treat, *IV* intravenous, *P* pertuzumab, *PH FDC SC* fixed-dose combination of pertuzumab and trastuzumab for subcutaneous injection, *PK* pharmacokinetic. ^a^Reasons for exclusion: five patients had missing C_trough_ pre-dose cycle 8 PK samples, three patients had their C_trough_ sample collected with ≥ 2 days deviation from the planned-on Day 21, two patients had a dose amount that deviated from the planned dose by > 20%, one patient had a dose delay of > 7 days, and one patient’s samples needed to be checked to see whether the pre-dose and post-dose samples were switched. ^b^Reasons for exclusion: five patients had missing C_trough_ pre-dose cycle 8 PK samples, three patients had their C_trough_ sample collected with ≥ 2 days deviation from the planned-on Day 21, three patients had a dose amount that deviated from the planned dose by > 20%, and one patient had a dose delay of > 7 days. ^c^Reasons for exclusion: four patients had missing C_trough_ pre-dose cycle 8 PK samples and six patients had their C_trough_ sample collected with ≥ 2 days deviation from the planned-on Day 21. ^d^Reasons for exclusion: four patients had missing C_trough_ pre-dose cycle 8 PK samples and six patients had their C_trough_ sample collected with ≥ 2 days deviation from the planned-on Day 21. ^e^Includes patients who withdrew due to the COVID-19 pandemic. ^f^Reasons include patient noncompliance, death, and progressive disease (after completing neoadjuvant treatment but prior to surgery)

Almost all patients (> 93% per arm) completed both the neoadjuvant phase and surgery (Fig. 2). Discontinuations were well balanced between the arms, with two patients from each discontinuing without undergoing surgery, and four discontinuing in the PH FDC SC arm and five in the P + H IV arm after surgery but before the adjuvant period.

Patient demographics and baseline characteristics were generally well balanced between arms (Table 1). There were numerically more overweight patients and patients with an ECOG PS of 0 in the P + H IV arm than in the PH FDC SC arm.

**Table 1:** Patient demographics and baseline characteristics (ITT population)

Demographic/disease characteristic	P + H IV (*n* = 101)	PH FDC SC (*n* = 99)	Total (*N* = 200)
Median age, years (range)	52.0 (27.0–74.0)	52.0 (27.0–69.0)	52.0 (27.0–74.0)
Median weight, kg (range)	61.0 (44.5–80.5)	60.0 (45.0–81.0)	61.0 (44.5–81.0)
BMI (WHO), *n* (%)			
Underweight (< 18.5 kg/m^2^)	3 (3)	2 (2)	5 (3)
Normal (18.5 to < 25.0 kg/m^2^)	55 (54)	63 (64)	118 (59)
Overweight (25.0 to < 30.0 kg/m^2^)	39 (39)	29 (29)	68 (34)
Obese (≥ 30 kg/m^2^)	4 (4)	5 (5)	9 (5)
Postmenopausal, *n* (%)	52 (51)	47 (47)	99 (50)
Baseline ECOG PS, *n* (%)			
0	95 (94)	83 (84)	178 (89)
1	6 (6)	16 (16)	22 (11)
Central HER2 IHC, *n* (%)			
2 +	5 (5)	7 (7)	12 (6)
3 +	96 (95)	91 (92)	187 (94)
NA	0	1 (1)	1 (< 1)
Central *HER2* ISH amplification, *n* (%)	101 (100)	99 (100)	200 (100)
HR status, *n* (%)			
ER- and/or PgR-positive	50 (50)	48 (48)	98 (49)
ER- and PgR-negative	51 (50)	51 (52)	102 (51)
Clinical stage at presentation, *n* (%)			
II–IIIA	71 (69)	70 (71)	140 (70)
IIIB–IIIC	30 (31)	29 (29)	60 (30)
Median time since initial diagnosis, weeks (range)	3.00 (1.1–9.7)	2.86 (1.1–8.6)	3.00 (1.1–9.7)
Primary tumor on right-hand side, *n* (%)	50 (50)	51 (52)	101 (51)
Histologic subtype, *n* (%)			
Invasive carcinoma of no special type	96 (95)	87 (88)	183 (92)
Invasive lobular carcinoma	0	2 (2)	2 (1)
Invasive micropapillary carcinoma	3 (3)	7 (7)	10 (5)
Histologic grade, *n* (%)			
GX	4 (4)	3 (3)	7 (4)
G1	0	1 (1)	1 (< 1)
G2	26 (26)	27 (27)	53 (27)
G3	14 (14)	10 (10)	24 (12)
No residual tumor	0	1 (1)	1 (< 1)
Unknown	57 (56)	57 (58)	114 (57)

*BMI* body mass index, *ECOG PS* Eastern Cooperative Oncology Group performance status, *ER* estrogen receptor, *H* trastuzumab, *HR* hormone receptor, *IHC* immunohistochemistry, *ISH* in situ hybridization, *ITT* intention-to-treat, *IV* intravenous, *P* pertuzumab, *PgR* progesterone receptor, *PH FDC SC* fixed-dose combination of pertuzumab and trastuzumab for subcutaneous injection, *WHO* World Health Organization

### Pharmacokinetics

The observed cycle 7 pertuzumab and trastuzumab serum C_trough_ (co-primary endpoints), area under the curve, and maximum concentration (C_max_) are shown in Table 2. The geometric mean for cycle 7 pertuzumab C_trough_ values was comparable between the PH FDC SC and P + H IV arms (74.6 μg/mL and 69.9 μg/mL, respectively), and the GMR (C_trough SC_/C_trough IV_) was 1.07 (90% CI 0.99–1.15). The geometric mean for cycle 7 trastuzumab C_trough_ values was higher in the PH FDC SC arm vs. the P + H IV arm (52.1 μg/mL vs. 33.6 μg/mL, respectively), and the GMR (C_trough SC_/C_trough IV_) was 1.55 (90% CI 1.44–1.67). The lower bounds of the GMR 90% CIs were both ≥ 0.8 (the specified non-inferiority margin), demonstrating non-inferior cycle 7 pertuzumab and trastuzumab serum C_trough_ for PH FDC SC compared with P + H IV. The mean cycle 7 pertuzumab and trastuzumab C_max_ was ~ 59% and ~ 28% higher, respectively, for P + H IV than for PH FDC SC.

**Table 2:** Observed P and H serum C_trough_, AUC, and C_max_ during cycle 7 (pre-dose cycle 8) (PK population)

	P		H	
	P + H IV (*n* = 89)	PH FDC SC (*n* = 89)	P + H IV (*n* = 89)	PH FDC SC (*n* = 89)
Serum C_trough_ (co-primary endpoint)				
Mean, μg/mL	73.0	77.8	34.9	54.8
Geometric mean, μg/mL	69.9	74.6	33.6	52.1
Median, μg/mL	69.5	74.8	34.0	52.1
Range, μg/mL	29.6–168	34.7–166	14.2–75.4	21.3–117.0
SD, μg/mL	22.5	23.7	10.0	17.8
CV, %	30.8	30.5	28.8	32.5
GMR (90% CI)	1.07 (0.99–1.15)		1.55 (1.44–1.67)	
AUC				
Geometric mean, μg/mL*day	2720	2400	1540	1810
GMR (90% CI)	0.88 (0.84–0.93)		1.17 (1.11–1.24)	
C_max_				
Mean, μg/mL	259	163	164	128

*AUC* area under the curve, *CI* confidence interval, *C*_*max*_ maximum concentration, *C*_*trough*_ trough concentration, *CV* coefficient of variation, *GMR* geometric mean ratio, *H* trastuzumab, *IV* intravenous, *P* pertuzumab, *PH FDC SC* fixed-dose combination of pertuzumab and trastuzumab for subcutaneous injection, *PK* pharmacokinetic, *SD* standard deviation

### Efficacy

tpCR rates in the PH FDC SC and P + H IV arms were 55.6% (95% CI 45.2–65.6) and 56.4% (95% CI 46.2–66.3), respectively, resulting in a tpCR difference between arms of –0.88% (95% CI −15.2 to 13.5) (Table 3). tpCR rates across age, clinical stage at presentation, histologic subtype, and histologic grade subgroups were consistent with the ITT population. tpCR rates were higher in the HR-negative subgroup vs. the HR-positive subgroup, and in the postmenopausal subgroup vs. premenopausal patients.

**Table 3:** tpCR rates (ITT population)

Subgroup	P + H IV (*n* = 101)			PH FDC SC (*n* = 99)			
	Patients, *n*	Responders, *n*	tpCR rate, % (95% CI)		Patients, *n*	Responders, *n*	tpCR rate, % (95% CI)
All	101	57	56.4 (46.2–66.3)		99	55	55.6 (45.2–65.6)
Age, years							
< 65	89	48	53.9 (43.0–64.6)		91	50	54.9 (44.2–65.4)
≥ 65	12	9	75.0 (42.8–94.5)		8	5	62.5 (24.5–91.5)
Clinical stage at presentation							
II–IIIA	70	40	57.1 (44.8–68.9)		70	38	54.3 (41.9–66.3)
IIIB–IIIC	31	17	54.8 (36.0–72.7)		29	17	58.6 (38.9–76.5)
HR status							
ER- and/or PgR-positive	50	23	46.0 (31.8–60.7)		48	22	45.8 (31.4–60.8)
ER- and PgR-negative	51	34	66.7 (52.1–79.2)		51	33	64.7 (50.1–77.6)
Histologic subtype							
Invasive carcinoma of no special type	96	53	55.2 (44.7–65.4)		87	49	56.3 (45.3–66.9)
Invasive micropapillary	3	3	100 (29.2–100)		7	3	42.9 (9.9–81.6)
Histologic grade							
GX	4	2	50.0 (6.8–93.2)		3	2	66.7 (9.4–99.2)
G2	26	13	50.0 (29.9–70.1)		27	13	48.1 (28.7–68.1)
G3	14	8	57.1 (28.9–82.3)		10	4	40.0 (12.2–73.8)
Unknown	57	34	59.6 (45.2–72.4)		57	35	61.4 (47.6–74.0)
Menopausal status							
Premenopausal	49	23	46.9 (32.5–61.7)		52	24	46.2 (32.2–60.5)
Postmenopausal	52	34	65.4 (50.9–78.0)		47	31	66.0 (50.7–79.1)

*CI* confidence interval, *ER* estrogen receptor, *H* trastuzumab, *HR* hormone receptor, *ITT* intention-to-treat, *IV* intravenous, *P* pertuzumab, *PgR* progesterone receptor, *PH FDC SC* fixed-dose combination of pertuzumab and trastuzumab for subcutaneous injection, *tpCR* total pathological complete response

### Exposure to HER2-targeted therapy

Exposure to HER2-targeted therapy was comparable between arms, with most patients in both arms completing all four scheduled cycles (Table 4).

**Table 4:** Exposure to HER2-targeted therapy (safety population)

HER2-targeted therapy exposure	P + H IV		PH FDC SC (*n* = 98)^a^
	P IV (*n* = 97)	H IV (*n* = 97)	
Completed assigned therapy, *n* (%)	95 (98)	95 (98)	95 (97)
Mean number of cycles (SD)	3.9 (0.4)	3.9 (0.4)	3.9 (0.5)
Median number of cycles (range)	4.0 (1–4)	4.0 (1–4)	4.0 (1–4)
Mean treatment duration, weeks (SD)	9.2 (1.3)	9.2 (1.3)	9.0 (1.5)
Median treatment duration, weeks (range)	9.1 (0–15)	9.1 (0–15)	9.1 (0–11)
Mean dose intensity, % (SD)	100 (0)	99.8 (3.6)	100 (0)
Patients with ≥ 1 dose delay, *n* (%)	13 (13)	13 (13)	7 (7)

*H* trastuzumab, *IV* intravenous, *P* pertuzumab, *PH FDC SC* fixed-dose combination of pertuzumab and trastuzumab for subcutaneous injection, *SD* standard deviation

^a^One patient who was randomized to the P + H IV arm mistakenly took PH FDC SC at cycle 5. This patient was assigned to the PH FDC SC arm in the safety population and thus only contributed to one cycle of PH FDC SC exposure

### Safety

A summary of AEs is given in Table 5. Diarrhea was more frequent in the P + H IV arm vs. the PH FDC SC arm, with most cases in each arm being grade 1–2. Infusion-/administration-related reactions occurred within 24 h in 12% and 14% of patients in the P + H IV and PH FDC SC arms, respectively; most cases were grade 1–2 (only one grade ≥ 3 event occurred with PH FDC SC). The most common AEs (occurring in ≥ 10% of patients in either arm) were anemia (65% for PH FDC SC vs. 63% for P + H IV), alopecia (66% vs. 58%), and neutrophil count decreased (60% vs. 60%) (Online Resource 1). The most common grade 3–4 AEs (occurring in ≥ 5% of patients in either arm) were neutrophil count decreased (48% vs. 45%), white blood cell count decreased (37% vs. 27%), and leukopenia (12% vs. 19%) (Online Resource 2). There was one cardiac death in the PH FDC SC arm, which was assessed by the investigators as being HER2-targeted therapy-related (Table 6).

**Table 5:** Summary of AEs (safety population)

Patients, %	P + H IV (*n* = 100)	PH FDC SC (*n* = 100)
Any AE	97	98
AE with fatal outcome	0	1
Grade ≥ 3 AE	69	72
Serious AE	19	18
Related serious AE (any)	16	16
AE leading to any study drug discontinuation	2	4
AE leading to HER2 therapy discontinuation	1	3
AE leading to chemotherapy discontinuation	1	2
AE leading to chemotherapy dose modification	8	14
Related AE	97	98
AE related to HER2-targeted therapy	90	90
AE related to chemotherapy	97	98
AE to monitor		
Anaphylaxis and hypersensitivity	1	4
Grade ≥ 3	0	1
IRR/ARR within 24 h	12	14
Grade ≥ 3	0	1
Serious rash/skin reactions	0	1
Grade ≥ 3	0	0
Diarrhea	39	27
Grade ≥ 3	6	0
Cardiac dysfunction	8	7
Grade ≥ 3	0	0
Interstitial lung disease	0	1
Grade ≥ 3	0	1
Neutropenia/febrile neutropenia^a^	75	75
Grade ≥ 3	61	65
Serious mucositis	0	1
Grade ≥ 3	0	1
Pregnancy- and neonatal-related	1	0
Grade ≥ 3	0	0

*AE* adverse event, *ARR* administration-related reaction, *H* trastuzumab, *IRR* injection-related reaction, *IV* intravenous, *P* pertuzumab, *PH FDC SC* fixed-dose combination of pertuzumab and trastuzumab for subcutaneous injection

^a^Includes white blood cell count decreased, neutrophil count decreased, febrile neutropenia, leukopenia, neutropenia, granulocyte count decreased, lymphocyte count decreased, monocyte count decreased, eosinophil count decreased, and eosinopenia

**Table 6:** Cardiac safety summary (safety population)

	P + H IV (*n* = 100)	PH FDC SC (*n* = 100)
Primary cardiac AE, patients, %	0	1
Heart failure (NYHA class III/IV) and significant LVEF decrease^a^	0	0
Cardiac death (definite or probable)	0	1
Secondary cardiac AE^b^, patients, %	1	4
Identified by initial LVEF assessments	1	4
Confirmed by second LVEF assessment	1	0
Mean baseline LVEF (SD)	66.4 (4.6)	66.2 (4.3)
Median baseline LVEF (range)	66.0 (58.0–84.0)	66.0 (58.0–78.0)
Mean change from baseline to worst value (SD)	− 5.4 (4.8)	− 5.2 (5.0)
Median change from baseline to worst value (range)	− 5.0 (− 18.8 to − 3.0)	− 5.0 (− 22.0 to − 3.6)
Treatment difference, SC–IV (95% CI)	0.29 (− 1.1 to 1.7)	
Worst value < 40%, patients, %	0	0
Patients with baseline value and ≥ 1 post-baseline treatment phase value, patients, %		
Increase or no change	16	20
Decrease of < 10%	65	64
Decrease of ≥ 10%	18	15
≤ 45% to < 50%	1.0	0
Drop in LVEF of ≥ 10% from baseline and to < 50%, patients, %	1	2
Asymptomatic decline in LVEF requiring treatment or leading to discontinuation of HER2-targeted therapy, patients, %	2	2
LVEF < 45% and decrease of ≥ 10% from baseline, patients, %	1	2

*AE* adverse event, *CI* confidence interval, *H* trastuzumab, *IV* intravenous, *LVEF* left ventricular ejection fraction, *NYHA* New York Heart Association, *P* pertuzumab, *PH FDC SC* fixed-dose combination of pertuzumab and trastuzumab for subcutaneous injection, *SD* standard deviation

^a^Significant LVEF decline defined as a drop in LVEF of ≥ 10% from baseline and to < 50%

^b^Drop in LVEF of ≥ 10% from baseline and to < 50%

## Discussion

PH FDC SC represents a treatment option that can benefit patients, society, and healthcare systems in terms of reduced treatment burden, increased patient productivity, and reduced medical resource utilization vs. P + H IV [7–12].

In our study, the co-primary endpoints of cycle 7 pertuzumab and trastuzumab serum C_trough_ (pre-dose cycle 8) were met and demonstrated non-inferiority of pertuzumab and trastuzumab within PH FDC SC vs. P + H IV. Non-inferiority of cycle 7 pertuzumab and trastuzumab serum C_trough_ for PH FDC SC vs. P + H IV was also shown in the global and Asian populations in the phase III FeDeriCa study [15]. The GMR of the cycle 7 pertuzumab serum C_trough_ in our study was 1.07 (90% CI 0.99–1.15), which is lower than that observed in both the global (GMR = 1.22 [90% CI 1.14–1.31]) and the Asian populations (GMR = 1.22 [90% CI 1.10–1.36]) in the FeDeriCa study [15, 17]. However, all patients receiving PH FDC SC in our study had cycle 7 pertuzumab serum C_trough_ values > 20 μg/mL, the target steady-state C_trough_ of pertuzumab derived from pre-clinical mouse xenograft models and used in early clinical response studies [18, 19].

The GMR of the cycle 7 trastuzumab serum C_trough_ in our study was 1.55 (90% CI 1.44–1.67), which was numerically higher than the GMR of 1.33 (90% CI 1.24–1.43) in the global population of the FeDeriCa study [15]. However, it was comparable to the GMR of 1.56 (90% CI 1.39–1.75) in the Asian subpopulation of the FeDeriCa study [17]. A possible explanation for the higher trastuzumab GMR observed in Chinese/Asian populations vs. the global population is the lower lean body weight in the former. Baseline body weight in the FDChina study and the FeDeriCa Asian subgroup was numerically lower than that in the global FeDeriCa population [15, 17], as expected. Of note, trastuzumab within PH FDC SC is administered as a fixed dose, whereas IV trastuzumab is administered as a weight-based dose. With weight-based dosing, the patients with lower lean body weight receive lower treatment doses and exposures, leading to a higher GMR. This was observed in patients in the P + H IV arm in our study (geometric mean = 33.6 μg/mL) and in the Asian subgroup patients in the P + H IV arm in FeDeriCa (geometric mean = 38.8 μg/mL) vs. patients in the P + H IV arm in the global FeDeriCa study (geometric mean = 43.2 μg/mL) [15, 17]. However, despite exposure to trastuzumab in the PH FDC SC arm in our study (geometric mean = 52.1 μg/mL) and in the FeDeriCa study (geometric mean = 57.5 μg/mL) [15] exceeding that in the P + H IV arm, trastuzumab’s well-defined safety profile and lack of exposure–response relationship [20] means that this increase in exposure in the PH FDC SC arm is unlikely to be clinically meaningful.

In the ITT population, ~56% of patients per arm achieved tpCR. These results are comparable to the tpCR rates observed in FeDeriCa [15] and to other pertuzumab studies, including TRYPHAENA, NeoSphere, KRISTINE, and BERENICE, where tpCR rates ranged from 54 to 64% [2, 21–23].

The overall safety profile in the PH FDC SC arm was comparable to that of the P + H IV arm, including for infusion-/administration-related reactions. This is in line with the expected toxicity profiles of regimens including pertuzumab and trastuzumab in combination with chemotherapy based on the results of previous studies [2, 15, 21, 23]. Grade ≥ 3 AEs occurred more frequently in FDChina (72% for PH FDC SC vs. 69% for P + H IV) than in FeDeriCa (49% vs. 53%) [15], which was mainly driven by differences in chemotherapy-related toxicity between the global and Chinese populations, and was related to differences in chemotherapy choice between the studies. The PERUSE study demonstrated a more favorable safety profile for paclitaxel and nab-paclitaxel compared with docetaxel [24]. In FeDeriCa, patients received either paclitaxel or docetaxel [15], while all patients in FDChina received docetaxel. Of note, the incidence of grade 3–4 AEs did not result in high premature withdrawal from HER2-targeted therapy, as evidenced by the high number of patients (≥ 95% in each arm) who had completed neoadjuvant treatment at the clinical cutoff date. This indicates that the administration of PH FDC SC with chemotherapy in Chinese patients is generally well tolerated and manageable.

Neutropenia/febrile neutropenia also occurred more frequently in FDChina (75% per arm) than in FeDeriCa (48% for PH FDC SC vs. 53% for P + H IV) [15]. This trend has been similarly observed in other studies that compare Asian subgroups with global patient populations (APHINITY Chinese vs. global patients [25], and FeDeriCa Asian vs. global patients [15, 17]). Although the reasons for this are not fully clear, differences in local practice for managing toxicities and differences in tolerability to chemotherapy in the Chinese/Asian populations are potential explanations. Importantly, the higher incidence of neutropenia/febrile neutropenia in FDChina did not lead to more treatment discontinuations or infections.

All-grade diarrhea occurred less frequently in FDChina (27% for PH FDC SC vs. 39% for P + H IV) than in FeDeriCa (59% vs. 55%) [15]. The majority of diarrhea AEs in FDChina were grade 1 or 2 in both arms. Grade 3 diarrhea was reported in the P + H IV arm only (6% of patients), of which 5% of patients had ≥ 1 HER2-targeted therapy-related diarrhea AE. No higher grade AEs were reported in either treatment arm, and there were no diarrhea serious AEs or AEs that led to withdrawal of any study medication.

The main strength of the FDChina study is that it was randomized and fully powered to evaluate the PK of both pertuzumab and trastuzumab in PH FDC SC vs. P + H IV specifically in Chinese patients with HER2-positive BC in the neoadjuvant–adjuvant setting. This was essential to assess the benefit of PH FDC SC in Chinese patients because this is an understudied population due to the low proportions of Asian patients in FeDeriCa, and because FeDeriaCa was not powered for racial subgroup analyses [15]. Importantly, the results support the non-inferiority of PH FDC SC vs. P + H IV in terms of cycle 7 (pre-dose cycle 8) serum C_trough_, as well as the comparable efficacy and safety profiles reported in the global FeDeriCa study [15].

This primary analysis of FDChina was performed after completion of the neoadjuvant phase before continuation of HER2-targeted therapy up to 18 cycles, per standard of care. Therefore, PK, safety, and efficacy results from the adjuvant phase and 3-year follow-up results are not yet available but will be published once these data are mature.

PH FDC SC was approved in China in December 2023 and has the potential to enable flexible care for patients with HER2-positive BC, which should help to reduce treatment burden for these patients [26]. In the randomized phase II PHranceSCa study, patients with HER2-positive eBC previously treated with neoadjuvant P + H IV and chemotherapy and surgery demonstrated a strong preference for PH FDC SC over P + H IV, with no new safety signals observed [27].

In conclusion, FDChina met its co-primary endpoints; cycle 7 serum C_trough_ for pertuzumab and trastuzumab for PH FDC SC were non-inferior to those for P + H IV. tpCR rates were comparable between arms and similar to previous pertuzumab-related studies in the neoadjuvant setting. The overall safety and tolerability profile of PH FDC SC was similar to that of P + H IV and consistent with the established pertuzumab and trastuzumab safety profiles in combination with chemotherapy, with no new or unexpected safety signals identified. PH FDC SC can offer a faster, more convenient, and less invasive treatment option than P + H IV. The favorable benefit–risk profile of PH FDC SC shown in this study suggests that PH FDC SC may be a viable treatment option for Chinese patients with HER2-positive eBC.

## Supplementary Information

Below is the link to the electronic supplementary material.

Supplementary file1 (DOCX 26 KB)

## Data Availability

Qualified researchers may request access to individual patient-level data through the clinical study data request platform: https://vivli.org/. Further details on Roche’s criteria for eligible studies are available here: https://vivli.org/members/ourmembers/. For further details on Roche’s Global Policy on the Sharing of Clinical Information and how to request access to related clinical study documents, see here: https://www.roche.com/research_and_development/who_we_are_how_we_work/clinical_trials/our_commitment_to_data_sharing.htm.
