# Supplementary material for: The fixed-dose combination of pertuzumab and trastuzumab for subcutaneous injection in Chinese patients with HER2-positive early breast cancer: primary analysis of the phase III, randomized FDChina study
Source: Int J Clin Oncol. 2026 May 7;31(7):1189–200. doi: 10.1007/s10147-025-02935-7 (PMC13303565; doi:10.1007/s10147-025-02935-7)
Supplement: Supplementary file 1 — Supplementary file1 (DOCX 26 KB) [file 10147_2025_2935_MOESM1_ESM.docx]

**Supplementary Information captions:**

**Online Resource 1** Most common AEs occurring in ≥ 10% of patients in either arm

**Online Resource 2** Most common grade 3–4 AEs occurring in ≥ 5% of patients in either arm

**Supplementary Information**

**The fixed-dose combination of pertuzumab and trastuzumab for subcutaneous injection in Chinese patients with HER2-positive early breast cancer: primary analysis of the phase III, randomized FDChina study**

***International Journal of Clinical Oncology***

Tao Huang · Zhimin Fan · Yongsheng Wang · Xi Yan · Hongjian Yang · Shu Wang · Da Pang · Huiping Li · Haibo Wang · Cuizhi Geng · Liang Huang · Yaqing Sun · Bei Wang · Guofang Sun · Asna Siddiqui · Eleonora Restuccia · Zhimin Shao

Corresponding author: Zhimin Shao, Breast Cancer, Fudan University Shanghai Cancer Center; [zhimingshao@fudan.edu.cn](mailto:zhimingshao@fudan.edu.cn)

**Online Resource 1** Most common AEs occurring in ≥ 10% of patients in either arm (safety population)

| MedDRA preferred term, % | P+H IV  (*n* = 100) | PH FDC SC  (*n* = 100) |
| --- | --- | --- |
| Anemia | 63 | 65 |
| Alopecia | 58 | 66 |
| Neutrophil count decreased | 60 | 60 |
| White blood cell count decreased | 47 | 56 |
| Nausea | 48 | 45 |
| Alanine aminotransferase increased | 42 | 46 |
| Vomiting | 42 | 46 |
| Aspartate aminotransferase increased | 40 | 38 |
| Diarrhea | 39 | 27 |
| Leukopenia | 25 | 17 |
| Platelet count decreased | 18 | 21 |
| Hypertriglyceridemia | 12 | 18 |
| Rash | 13 | 16 |
| Lymphocyte count decreased | 13 | 14 |
| Malaise | 15 | 12 |
| Hypoalbuminemia | 13 | 13 |
| Pyrexia | 15 | 11 |
| Mouth ulceration | 11 | 14 |
| Decreased appetite | 13 | 11 |
| Constipation | 10 | 12 |
| Blood alanine phosphatase increased | 9 | 12 |
| Cough | 8 | 13 |
| Hyperuricemia | 10 | 11 |

*AE* adverse event, *H* trastuzumab, *IV* intravenous, *MedDRA* Medical Dictionary for Regulatory Activities, *P* pertuzumab, *PH FDC SC* fixed-dose combination of pertuzumab and trastuzumab for subcutaneous injection

**The fixed-dose combination of pertuzumab and trastuzumab for subcutaneous injection in Chinese patients with HER2-positive early breast cancer: primary analysis of the phase III, randomized FDChina study**

***International Journal of Clinical Oncology***

Tao Huang · Zhimin Fan · Yongsheng Wang · Xi Yan · Hongjian Yang · Shu Wang · Da Pang · Huiping Li · Haibo Wang · Cuizhi Geng · Liang Huang · Yaqing Sun · Bei Wang · Guofang Sun · Asna Siddiqui · Eleonora Restuccia · Zhimin Shao

Corresponding author: Zhimin Shao, Breast Cancer, Fudan University Shanghai Cancer Center; [zhimingshao@fudan.edu.cn](mailto:zhimingshao@fudan.edu.cn)

**Online Resource 2** Most common grade 3–4 AEs occurring in ≥ 5% of patients in either arm (safety population)

| MedDRA preferred term, % | P+H IV  (*n* = 100) | PH FDC SC  (*n* = 100) |
| --- | --- | --- |
| Neutrophil count decreased | 45 | 48 |
| White blood cell count decreased | 27 | 37 |
| Leukopenia | 19 | 12 |
| Neutropenia | 7 | 8 |
| Febrile neutropenia | 4 | 10 |
| Lymphocyte count decreased | 7 | 3 |
| Anemia | 3 | 5 |
| Diarrhea | 6 | 0 |

*AE* adverse event, *H* trastuzumab, *IV* intravenous, *MedDRA* Medical Dictionary for Regulatory Activities, *P* pertuzumab, *PH FDC SC* fixed-dose combination of pertuzumab and trastuzumab for subcutaneous injection
